# Supplementary material for: Cell-selective proteomics reveal novel effectors secreted by an obligate intracellular bacterial pathogen
Source: Nat Commun. 2024 Jul 18;15:6073. doi: 10.1038/s41467-024-50493-9 (PMC11258249; doi:10.1038/s41467-024-50493-9)
Supplement: Supplementary file 1 — Supplementary Information [file 41467_2024_50493_MOESM1_ESM.pdf]

Supplementary Information

Cell-selective proteomics reveal novel effectors secreted by an obligate intracellular bacterial pathogen

Allen G. Sanderlin[1], Hannah Kurka Margolis[1], Abigail F. Meyer[1], Rebecca L. Lamason[1,*]

[1]Department of Biology, Massachusetts Institute of Technology, Cambridge, Massachusetts, USA

*Corresponding author, rlamason@mit.edu

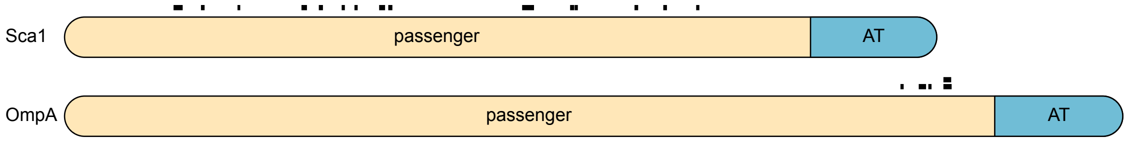

Supplementary Fig. 1 | Tryptic peptides mapping to autotransporter proteins Sca1 and OmpA.
Positions of unique peptides (black boxes) and passenger and autotransporter (AT) domains are indicated.

a

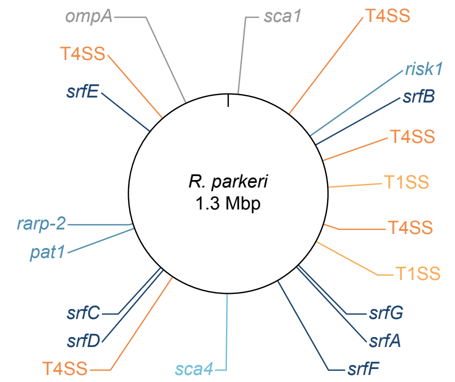

Supplementary Fig. 2 | Genomic positions and gene neighborhoods of *srf* loci.
a *R. parkeri* genome map displaying loci encoding SrfA–G; autotransporter proteins Sca1 and OmpA; known secreted effectors RARP-2, Pat1, Risk1, and Sca4; and components of the type IV (T4SS: RvhBD) and putative type I (T1SS: TolC, AprDE) secretion systems.
b Gene neighborhoods for *srfA–G* (black) across select members of the *Rickettsia* genus. Shared flanking genes are colored if found in at least three of the aligned genomes, and continuous gene neighborhoods for *srfG* and *srfA* and for *srfD* and *srfC* are highlighted for clarity. The putative *srfE* homolog of *R. bellii* (*RBE_1196*) is located distal to the flanking genes shared by other species and is therefore absent from the alignment. hp, hypothetical protein; *pa*, *R. parkeri*; *ri*, *R. rickettsii*; *Ip*, *Rickettsia* endosymbiont of *Ixodes pacificus*; *fe*, *R. felis*; *ty*, *R. typhi*; *ca*, *R. canadensis*; *be*, *R. bellii*. Scale bar, 1 kbp.

b

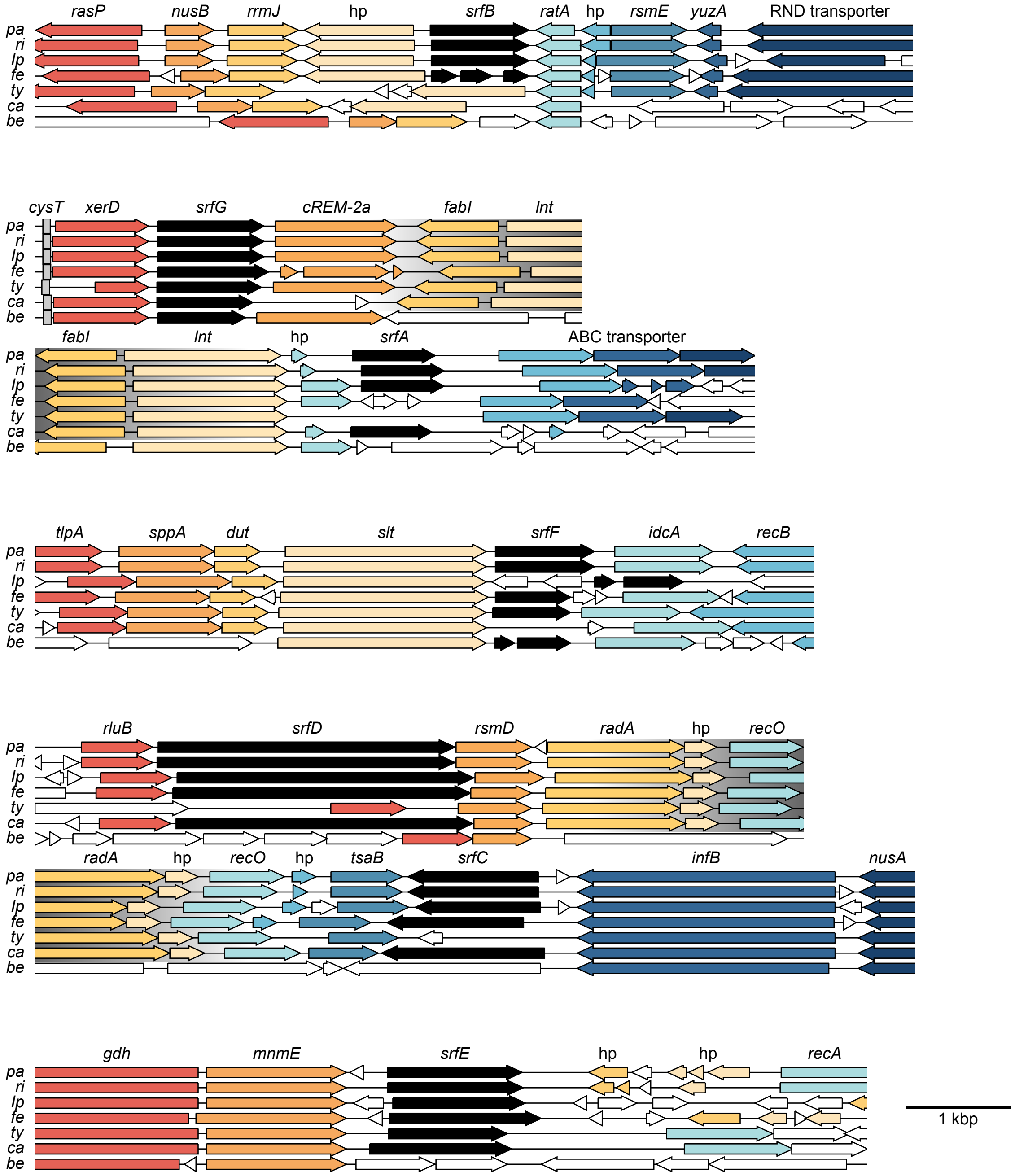

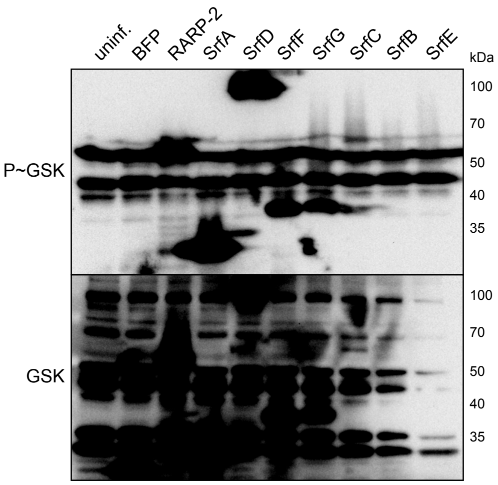

Supplementary Fig. 3 | GSK-tagged SrfB and SrfE are not obviously expressed by *R. parkeri*.
Western blots from **Fig. 3a** with enhanced contrast. SrfB and SrfE (expected 37 and 50 kDa, respectively) were not detected. Source data are provided as a Source Data file.

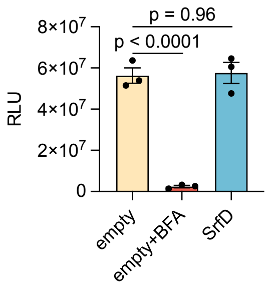

Supplementary Fig. 4 | SrfD does not impact secretion of *Gaussia* luciferase.
HEK293T cells stably expressing *Gaussia* luciferase were transiently transfected in triplicate (n = 3) with either empty vector or 3xFLAG-tagged SrfD and treated with DMSO or brefeldin A (BFA) prior to measuring luciferase activity of the culture supernatants. Relative light units (RLU) from a representative experiment were used to calculate the means ± SD and p-values (one-way ANOVA with post hoc Dunnett's test, F = 74.15, q = 10.42 and 0.24, df = 6). Results are representative of two independent experiments. Source data are provided as a Source Data file.

Strain or plasmid	Genotype or feature	Reference or source
***R. parkeri* strains**		
R. parkeri str. Portsmouth	Parental *R. parkeri* strain	Chris Paddock
WT	pRAM18dSGA[MCS]	This study
MetRS*	pRL0128	This study
GSK-BFP	pRL0284	1
GSK-RARP-2	pRL0285	1
GSK-SrfA	pRL0368	This study
GSK-SrfB	pRL0369	This study
GSK-SrfC	pRL0370	This study
GSK-SrfD	pRL0371	This study
GSK-SrfE	pRL0372	This study
GSK-SrfF	pRL0373	This study
GSK-SrfG	pRL0374	This study
**Plasmids**		
pRAM18dSGA[MCS]	*Rickettsia* shuttle vector	Ulrike Munderloh
pRL0128	MetRS*	This study
pRL0284	GSK-tagged TagBFP	1
pRL0285	GSK-tagged RARP-2	1
pRL0368	GSK-tagged SrfA	This study
pRL0369	GSK-tagged SrfB	This study
pRL0370	GSK-tagged SrfC	This study
pRL0371	GSK-tagged SrfD	This study
pRL0372	GSK-tagged SrfE	This study
pRL0373	GSK-tagged SrfF	This study
pRL0374	GSK-tagged SrfG	This study
pRL0375	GST-tagged SrfC	This study
pRL0376	GST-tagged SrfD(Δ766–957)	This study
pRL0377	GST-tagged SrfF	This study
pRL0378	6xHis-SUMO-TwinStrep-tagged SrfD(Δ766–957)	This study
pRL0379	6xHis-SUMO-TwinStrep-tagged SrfF	This study
pRL0430	6xHis-MBP-tagged SrfE(277–403)	Supratim Dey & Karla Satchell
pRL0381	empty vector control	This study
pRL0382	3xFLAG-tagged SrfA	This study
pRL0383	3xFLAG-tagged SrfB	This study
pRL0384	3xFLAG-tagged SrfC	This study
pRL0385	3xFLAG-tagged SrfD	This study
pRL0386	3xFLAG-tagged SrfE	This study
pRL0387	3xFLAG-tagged SrfF	This study
pRL0388	3xFLAG-tagged SrfG	This study
FCW2IB-BiP-mNeonGreen-KDEL	BiP(1–18)-mNeonGreen-KDEL	2
pRL0389	*Gaussia*-Dura luciferase	This study
pRL0390	3xFLAG-tagged SrfDΔPPR1	This study
pRL0391	3xFLAG-tagged SrfDΔCC1	This study
pRL0392	3xFLAG-tagged SrfDΔPPR2	This study
pRL0393	3xFLAG-tagged SrfDΔTM	This study
pRL0394	3xFLAG-tagged SrfDΔCC2	This study

Supplementary Table 1. Strains and plasmids used in this study.

Supplementary References

1. Sanderlin, A. G., Hanna, R. E. & Lamason, R. L. The ankyrin repeat protein RARP-1 is a periplasmic factor that supports Rickettsia parkeri growth and host cell invasion. *J. Bacteriol.* **204**, e00182-22 (2022).
2. Acevedo-Sánchez, Y., Woida, P. J., Kraemer, S. & Lamason, R. L. An obligate intracellular bacterial pathogen forms a direct, interkingdom membrane contact site. Preprint at https://doi.org/10.1101/2023.06.05.543771 (2023).
