# Supplementary material for: Cell-selective proteomics reveal novel effectors secreted by an obligate intracellular bacterial pathogen
Source: Nat Commun. 2024 Jul 18;15:6073. doi: 10.1038/s41467-024-50493-9 (PMC11258249; doi:10.1038/s41467-024-50493-9)
Supplement: Supplementary file 3 — Description of Additional Supplementary Files [file 41467_2024_50493_MOESM3_ESM.pdf]

Description of Additional Supplementary Files

File Name: Supplementary Data 1
Description: BONCAT pull-down mass spectrometry results.

File Name: Supplementary Data 2
Description: SrfD co-immunoprecipitation mass spectrometry results.
