# Supplementary material for: Cell-selective proteomics reveal novel effectors secreted by an obligate intracellular bacterial pathogen
Source: Nat Commun. 2024 Jul 18;15:6073. doi: 10.1038/s41467-024-50493-9 (PMC11258249; doi:10.1038/s41467-024-50493-9)
Supplement: Supplementary file 6 — Reporting Summary [file 41467_2024_50493_MOESM6_ESM.pdf]

nature portfolio

Corresponding author(s): Rebecca L. Lamason

Last updated by author(s): Jun 26, 2024

Reporting Summary

Nature Portfolio wishes to improve the reproducibility of the work that we publish. This form provides structure for consistency and transparency in reporting. For further information on Nature Portfolio policies, see our Editorial Policies and the Editorial Policy Checklist.

Statistics

For all statistical analyses, confirm that the following items are present in the figure legend, table legend, main text, or Methods section.

n/a	Confirmed	
☐	☒	The exact sample size (*n*) for each experimental group/condition, given as a discrete number and unit of measurement
☐	☒	A statement on whether measurements were taken from distinct samples or whether the same sample was measured repeatedly
☐	☒	The statistical test(s) used AND whether they are one- or two-sided<br>*Only common tests should be described solely by name; describe more complex techniques in the Methods section.*
☒	☐	A description of all covariates tested
☐	☒	A description of any assumptions or corrections, such as tests of normality and adjustment for multiple comparisons
☐	☒	A full description of the statistical parameters including central tendency (e.g. means) or other basic estimates (e.g. regression coefficient) AND variation (e.g. standard deviation) or associated estimates of uncertainty (e.g. confidence intervals)
☐	☒	For null hypothesis testing, the test statistic (e.g. *F*, *t*, *r*) with confidence intervals, effect sizes, degrees of freedom and *P* value noted<br>*Give P values as exact values whenever suitable.*
☒	☐	For Bayesian analysis, information on the choice of priors and Markov chain Monte Carlo settings
☒	☐	For hierarchical and complex designs, identification of the appropriate level for tests and full reporting of outcomes
☒	☐	Estimates of effect sizes (e.g. Cohen's *d*, Pearson's *r*), indicating how they were calculated

Our web collection on statistics for biologists contains articles on many of the points above.

Software and code

Policy information about availability of computer code

Data collection	cellSens Dimension 2.2 (Olympus) was used to acquire microscopy images. Image Lab Touch 2.3 (Bio-Rad) was used to acquire blot images. SkanIt RE 2.4.5 (Thermo Fisher Scientific) was used to acquire luciferase assay data.
Data analysis	FIJI/ImageJ 2.1.0/1.53c was used to analyze images. PEAKS Studio 10.6 (Bioinformatics Solutions), Proteome Discoverer 2.5 (Thermo Fisher Scientific), and Scaffold 5.0.1 (Proteome Software) were used to analyze mass spectrometry data. Prism 9 and 10 (GraphPad Software) were used for statistical analysis.

For manuscripts utilizing custom algorithms or software that are central to the research but not yet described in published literature, software must be made available to editors and reviewers. We strongly encourage code deposition in a community repository (e.g. GitHub). See the Nature Portfolio guidelines for submitting code & software for further information.

Data

Policy information about availability of data

All manuscripts must include a data availability statement. This statement should provide the following information, where applicable:

- Accession codes, unique identifiers, or web links for publicly available datasets
- A description of any restrictions on data availability
- For clinical datasets or third party data, please ensure that the statement adheres to our policy

Source data are provided with this paper. The protein mass spectrometry data generated in this study have been deposited in the public proteomics repository MassIVE (https://massive.ucsd.edu) under accession codes MSV000093380 and MSV000093381.

Research involving human participants, their data, or biological material

Policy information about studies with human participants or human data. See also policy information about sex, gender (identity/presentation), and sexual orientation and race, ethnicity and racism.

Reporting on sex and gender	*Use the terms sex (biological attribute) and gender (shaped by social and cultural circumstances) carefully in order to avoid confusing both terms. Indicate if findings apply to only one sex or gender; describe whether sex and gender were considered in study design; whether sex and/or gender was determined based on self-reporting or assigned and methods used. Provide in the source data disaggregated sex and gender data, where this information has been collected, and if consent has been obtained for sharing of individual-level data; provide overall numbers in this Reporting Summary. Please state if this information has not been collected. Report sex- and gender-based analyses where performed, justify reasons for lack of sex- and gender-based analysis.*
Reporting on race, ethnicity, or other socially relevant groupings	*Please specify the socially constructed or socially relevant categorization variable(s) used in your manuscript and explain why they were used. Please note that such variables should not be used as proxies for other socially constructed/relevant variables (for example, race or ethnicity should not be used as a proxy for socioeconomic status). Provide clear definitions of the relevant terms used, how they were provided (by the participants/respondents, the researchers, or third parties), and the method(s) used to classify people into the different categories (e.g. self-report, census or administrative data, social media data, etc.) Please provide details about how you controlled for confounding variables in your analyses.*
Population characteristics	*Describe the covariate-relevant population characteristics of the human research participants (e.g. age, genotypic information, past and current diagnosis and treatment categories). If you filled out the behavioural & social sciences study design questions and have nothing to add here, write "See above."*
Recruitment	*Describe how participants were recruited. Outline any potential self-selection bias or other biases that may be present and how these are likely to impact results.*
Ethics oversight	*Identify the organization(s) that approved the study protocol.*

Note that full information on the approval of the study protocol must also be provided in the manuscript.

Field-specific reporting

Please select the one below that is the best fit for your research. If you are not sure, read the appropriate sections before making your selection.

☒ Life sciences ☐ Behavioural & social sciences ☐ Ecological, evolutionary & environmental sciences

For a reference copy of the document with all sections, see nature.com/documents/nr-reporting-summary-flat.pdf

Life sciences study design

All studies must disclose on these points even when the disclosure is negative.

Sample size	No statistical methods were used to predetermine sample size. Samples sizes were determined based on the number of samples that could be processed in the given time frame or were otherwise previously determined to be sufficient to detect differences between conditions in similar experiments from the following publications: Sanderlin et al., J Bacteriol 2022 (doi: 10.1128/jb.00182-22) and Lamason et al., Cell 2016 (doi: 10.1016/j.cell.2016.09.023).
Data exclusions	For analysis of the SrfD co-immunoprecipitation mass spectrometry results, protein abundances were filtered to require a non-zero value for at least two of the three replicates in at least one condition so as to exclude any proteins that were sparsely quantified from comparisons between conditions.
Replication	At least 2 to 3 independent replicates were completed per experiment and gave reproducible results, as indicated in the figure legends. The BONCAT pull-down mass spectrometry results and SrfD co-IP mass spectrometry results reproduced the results from earlier pilot experiments.
Randomization	Samples were randomly allocated into experimental groups (e.g., infected/uninfected, +/- Anl).

Blinding	When possible, other lab members were given the blinded data and asked to quantify the data to confirm equivalent results to those reported here.

Reporting for specific materials, systems and methods

We require information from authors about some types of materials, experimental systems and methods used in many studies. Here, indicate whether each material, system or method listed is relevant to your study. If you are not sure if a list item applies to your research, read the appropriate section before selecting a response.

Materials & experimental systems

n/a	Involved in the study
☐	☒ Antibodies
☐	☒ Eukaryotic cell lines
☒	☐ Palaeontology and archaeology
☒	☐ Animals and other organisms
☒	☐ Clinical data
☒	☐ Dual use research of concern
☒	☐ Plants

Methods

n/a	Involved in the study
☒	☐ ChIP-seq
☒	☐ Flow cytometry
☒	☐ MRI-based neuroimaging

Antibodies

Antibodies used	Antibodies for β-catenin (mouse, clone L54E2, 2677S, 1:100 dilution), GSK-3β-Tag (rabbit, 9325S, 1:1000 dilution), phospho-GSK-3β (rabbit, 9336S, 1:1000 dilution), AIF (rabbit, clone D39D2, 5318S, 1:200 dilution), and Sec61β (rabbit, clone D5Q1W, 14648S, 1:200/1:4000/1:100 dilution [immunofluorescence/immunoblot/immunoprecipitation]) were purchased from Cell Signaling Technology. Mouse anti-RpoA (clone 4RA2, 663104, 1:5000 dilution) was purchased from BioLegend. Mouse anti-FLAG (clone M2, F1804, 1:400/1:4000 dilution [immunofluorescence/immunoblot]) was purchased from Sigma-Aldrich. StrepTactin-HRP (1610381, 1:12000 dilution) was purchased from Bio-Rad. Mouse (14-13, 1:400 dilution) and rabbit (I7205, 1:1000 dilution) anti-Rickettsia antibodies were gifts from Ted Hackstadt. Rabbit anti-SrfC (1:250/1:2000 dilution [immunofluorescence/immunoblot]), anti-SrfD (1:400/1:2000/15 ug/mL dilution [immunofluorescence/immunoblot/immunoprecipitation]), anti-SrfE (1:400/1:2000 dilution [immunofluorescence/immunoblot]), and anti-SrfF (1:400/1:2000 dilution [immunofluorescence/immunoblot]) antibodies were generated and affinity purified as described in this study.
Validation	All commercial antibodies were selected based on thorough literature searches for publications demonstrating well-controlled use of the antibodies. Anti-β-catenin (RRID: AB_1030943) was used previously (e.g., in Borgo et al., Nat Commun 2022 [doi: 10.1038/s41467-022-31351-y] and Lamason et al., Cell 2016 [doi: 10.1016/j.cell.2016.09.023]) and was validated for immunofluorescence microscopy and other applications as described by the manufacturer on their website. Anti-GSK-3β-Tag (RRID: AB_823513) and anti-phospho-GSK-3β (RRID: AB_331405) were used previously (e.g., in Sanderlin et al., J Bacteriol 2022 [doi: 10.1128/jb.00182-22] and Lehman et al., mBio 2018 [doi: 10.1128/mBio.00975-18]) and were validated for blotting as described by the manufacturer on their website. Anti-AIF (RRID: AB_10634755) was used previously (e.g., in Girardi et al., Nat Commun 2020 [doi: 10.1038/s41467-020-19871-x] and Ding et al., Nucleic Acids Research 2018 [doi: 10.1093/nar/gky1281]) and was validated for immunofluorescence microscopy and other applications as described by the manufacturer on their website. Anti-Sec61β (RRID: AB_2798555) was used previously (e.g., in Yamada et al., PNAS 2022 [doi: 10.1073/pnas.2202730119] and Leonetti et al., PNAS 2016 [doi: 10.1073/pnas.1606731113]) and was validated for immunofluorescence microscopy, blotting, and immunoprecipitation as described by the manufacturer on their website. Anti-RpoA (RRID: AB_2687386) was used previously (e.g., in Sanderlin et al., J Bacteriol 2022 [doi: 10.1128/jb.00182-22] and Guzzo et al., Dev Cell 2021 [doi: 10.1016/j.devcel.2021.06.014]) and validated for blotting as described by the manufacturer on their website. Anti-FLAG (RRID: AB_262044) was used previously (e.g., in Eubelen et al., Science 2018 [doi: 10.1126/science.aat1178] and Lamason et al., Cell 2016 [doi: 10.1016/j.cell.2016.09.023]) and validated for immunofluorescence microscopy, blotting, and other applications as described by the manufacturer on their website. StrepTactin-HRP was used previously (e.g., in Yang et al., Nat Commun 2017 [doi: 10.1038/s41467-017-02409-z]) and tested by the manufacturer. Anti-Rickettsia antibodies were used previously (e.g., in Sanderlin et al., J Bacteriol 2022 [doi: 10.1128/jb.00182-22] and Engström et al., Nat Microbiol 2019 [doi: 10.1038/s41564-019-0583-6]). Anti-SrfC, anti-SrfD, anti-SrfE, and anti-SrfF antibodies were validated in this study by blotting against purified R. parkeri, uninfected A549 cell lysates, and purified recombinant Srfs. Anti-SrfD was further validated in this study by immunoprecipitation mass spectrometry (Fig. 5a and Supplementary Data 2).

Eukaryotic cell lines

Policy information about cell lines and Sex and Gender in Research

Cell line source(s)	A549 (human lung epithelial), HeLa (human cervical epithelial), HEK293T (human embryonic kidney epithelial), and Vero (monkey kidney epithelial) cell lines were obtained from the University of California, Berkeley Cell Culture Facility (Berkeley, CA).
Authentication	Validation of cell lines is managed at the UCB Cell Culture Facility.
Mycoplasma contamination	All cell lines tested negative for mycoplasma contamination.

Commonly misidentified lines (See ICLAC register)	No misidentified lines were used.

Plants

Seed stocks	*Report on the source of all seed stocks or other plant material used. If applicable, state the seed stock centre and catalogue number. If plant specimens were collected from the field, describe the collection location, date and sampling procedures.*
Novel plant genotypes	*Describe the methods by which all novel plant genotypes were produced. This includes those generated by transgenic approaches, gene editing, chemical/radiation-based mutagenesis and hybridization. For transgenic lines, describe the transformation method, the number of independent lines analyzed and the generation upon which experiments were performed. For gene-edited lines, describe the editor used, the endogenous sequence targeted for editing, the targeting guide RNA sequence (if applicable) and how the editor was applied.*
Authentication	*Describe any authentication procedures for each seed stock used or novel genotype generated. Describe any experiments used to assess the effect of a mutation and, where applicable, how potential secondary effects (e.g. second site T-DNA insertions, mosiacism, off-target gene editing) were examined.*
